# Nucleotide sequencing of the HoxA gene cluster using Gorilla fosmid clones

**DOI:** 10.7150/jgen.50468

**Published:** 2020-08-29

**Authors:** Takashi Kitano, Choong-Gon Kim, Naruya Saitou

**Affiliations:** Division of Population Genetics, National Institute of Genetics, Japan.

**Keywords:** Fosmid library, Gorilla, HoxA

## Abstract

We sequenced the western gorilla (*Gorilla gorilla*) HoxA cluster region using seven fosmid clones, and found that the total tiling path sequence was 214,185 bp from the 5' non-genic region of HoxA1 to the 3' non-genic region of Evx1. We compared the nucleotide sequence with the gorilla genome sequence in the NCBI database, and the overall proportion of nucleotide difference was estimated to be 0.0005-0.0007. These estimates are lower than overall genomic polymorphism in gorillas.

## Introduction

The euchromatic sequence of the human genome was sequenced using the hierarchical shotgun sequencing strategy, also known as clone-by-clone sequencing, by the International Human Genome Sequencing Consortium 1. With the advent of next-generation sequencer (NGS), the hierarchical shotgun sequencing strategy is now less commonly used. However, hierarchical shotgun sequencing involving bacterial artificial chromosomes (BACs) and fosmids is still used for some purposes, such as to determine the long complete haploid in a chromosome region.

Kim et al. 2 constructed a western gorilla (*Gorilla gorilla*) fosmid library and established a simple polymerase chain reaction (PCR) screening system for it. They also selected seven fosmid clones, which constitutes the minimum tiling path for the entire HoxA gene cluster in the gorilla genome 2. In this study we sequenced these fosmid clones and used them in our analysis.

## Materials and Methods

Seven fosmid clones originated from one female gorilla individual “Taiko” (GGFP-562J15, GGFP-367A20, GGFP-347D05, GGFP-175G07, GGFP-452O13, GGFP-012E07, and GGFP-210K06) were screened and selected in a previous study 2. These seven clones were used for this study. We determined nucleotide sequences primarily using the hierarchical shotgun sequencing method, as previously described 3, by sequencing each clone with more than tenfold-coverage. For base-calling, assembly, and to obtain a quality score for both raw and assembled data we used the Phred-Phrap software package 4. Editing was performed with Consed 5. Finishing was carried out by primer walking and PCR-coupled primer walking.

The fosmid sequences were compared with the nucleotide sequences (assembly NC_044609.1 from the Kamilah_GGO_v0 genome) available in the National Center for Biotechnology Information (NCBI) database. Pairwise sequence alignment was done manually using MEGA7 software 6. Transitions, transversions, synonymous and nonsynonymous substitutions were counted using this software. Synonymous and nonsynonymous substitutions were estimated using Nei and Gojobori's method 7. When counting substitutions, the cluster sequence was divided into coding sequence (CDS), intron, and inter-genic regions. The 53 amino acid residues (e.g. RTNFTTKQLTELEKEFHFNKYLTRARRVEIAASLQLNETQVKIWFQNRRMKQK from HOXA1) were used as the homeodomain regions.

## Results and Discussion

**Figure 1:**
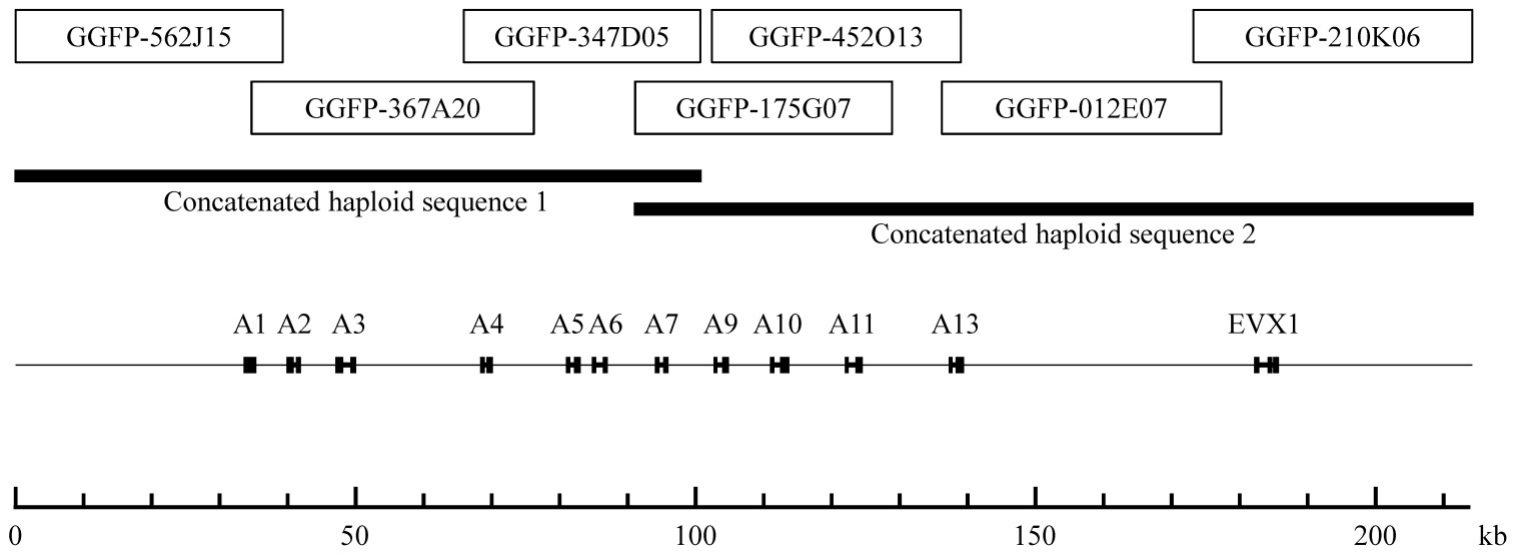
Mapping of the seven fosmid clones that constitute the minimum tiling path for the entire HoxA gene cluster of the gorilla genome. The seven fosmid clones are indicated by white boxes. The two concatenated haploid sequences are indicated by thick bars. The CDS regions of the HoxA genes and the EVX1 gene are indicated by black boxes.

The nucleotide sequences of the seven fosmid clones are as follows: GGFP-562J15 (39,323 bp), GGFP-367A20 (41,479 bp), GGFP-347D05 (34,811 bp), GGFP-175G07 (37,837 bp), GGFP-452O13 (36,576 bp), GGFP-012E07 (41,057 bp), and GGFP-210K06 (40,950 bp) (**Figure 1**). We found four identical overlapping regions: 4,635 bp of nucleotides between GGFP-562J15 (AB125652) and GGFP-367A20 (AB125653); 10,316 bp of nucleotides between GGFP-367A20 (AB125653) and GGFP-347D05 (AB125654); 2,819 bp of nucleotides between GGFP-452O13 (AB125656) and GGFP-012E07 (AB125657); and 3,986 bp of nucleotides between GGFP-012E07 (AB125657) and GGFP-210K06 (AB125658).

The overlap region between GGFP-175G07 (AB125655) and GGFP-452O13 (AB125656) had one indel in 26,513 bp. We used PCR to sequence this region using the genomic DNA of the gorilla (Taiko), and we confirmed the probable loss of one base in GGFP-452O13 (AB125656). The nucleotides in the overlap region between GGFP-347D05 (AB125654) and GGFP-175G07 (AB125655) differed by 0.09% (9/9,560 bp). We therefore linked and concatenated GGFP-562J15, GGFP-367A20, and GGFP-347D05 as “concatenated haploid sequence 1” (100,662 bp) and GGFP-175G07, GGFP-452O13, GGFP-012E07, and GGFP-210K06 as “concatenated haploid sequence 2” (123,103 bp) (**Figure 1**). The nucleotide difference of 0.09% between GGFP-347D05 (AB125654) and GGFP-175G07 (AB125655) can be considered the difference between maternal and paternal chromosomes.

We compared the nucleotide difference (*p*-distance) between our two concatenated haploid gorilla fosmid sequences and the whole-genome shotgun sequence of the gorilla (assembly NC_044609.1 from the Kamilah_GGO_v0 genome) (**Table 1**). The overall nucleotide difference in the HoxA cluster region between concatenated haploid sequence 1 and the shotgun sequence (100,577 bp) was 0.0005, and between concatenated haploid sequence 2 and the shotgun sequence (123,061 bp) was 0.0008. These estimates were lower than the genomic polymorphisms reported from two other western gorillas [Kamilah: 0.189% (0.00189), EB(JC): 0.178% (0.00178)] 8. These results are reasonable, because nucleotide differences in the HoxA gene cluster region are expected to be lower than in other genomic regions.

In the intron regions, the average *p*-distance was 0.0007, ranging from 0 [in HoxA1, HoxA2, HoxA7 (in concatenated haploid sequence 2)], HoxA9, HoxA13, and Evx1) to 0.0021 (in HoxA5).

In the CDS regions, the average *p*-distance was 0.0004, ranging from 0 [in HoxA1, HoxA3, HoxA6, HoxA7 (in concatenated haploid sequence 1), HoxA7 (in concatenated haploid sequence 2), HoxA11, HoxA13, and Evx1] to 0.0012 (in HoxA5 and HoxA9). One synonymous difference was observed in HoxA2. One nonsynonymous difference was observed for each of HoxA4, HoxA5, and HoxA9, although these changes were not located in homeobox regions.

An 18 bp gap was observed in exon 1 of HoxA10. The fosmid sequences were 18 bp longer than the whole-genome shotgun sequence of the gorilla (assembly NC_044609.1 from the Kamilah_GGO_v0 genome). When we examined the same region in humans (*Homo sapiens*), chimpanzees (*Pan troglodytes*), and orangutans (*Pongo abelii*), we found that they did not contain the 18 bp insertion. We used PCR to sequence this region in the DNA of the gorilla (Taiko) genome and confirmed that Taiko did have the 18 bp insertion. Because this insertion was observed in the non-homeobox region, we hypothesize that this insertion does not affect the fundamental function of HoxA10.

In the intergenic regions, the average *p*-distance was 0.0007, ranging from 0 [in the intergenic A1-A2 and A6-A7 (in concatenated haploid sequence 2)] to 0.0012 [in the intergenic A4-A5, A7-A9 (in concatenated haploid sequence 1), A10-A11, and A13-EVX1]. The differences observed in these intergenic regions were comparable with those observed in the intron regions.

The estimates for the CDS, introns, and intergenic regions were lower than the genomic polymorphisms reported from two other western gorillas (Kamilah: 0.00189, EB(JC): 0.00178) 8. It has been previously reported that the four Hox gene clusters have the lowest density of interspersed repeats in the human genome 9, probably because of the large-scale *cis*-regulatory elements that cannot tolerate being interrupted by insertions 10-12. The lower estimates we observed in the HoxA gene cluster region are therefore reasonable.

In conclusion, we successfully sequenced the HoxA cluster region of the gorilla. This region consists of two stretched haploid sequences that will be available for further analysis, including *cis*-regulatory element, linkage disequilibrium, and recombination.

## Figures and Tables

**Table 1:** Nucleotide differences in the HoxA gene cluster sequences between two gorillas

Region	Bp compared	ts	tv	ts+tv	*p*-distance	*s*	n*
**Concatenated haploid sequence 1 (AB125652-AB125654) vs. NC_044609.1**							
5p non-genic	33,704	6	3	9	0.0003		
A1 CDS	1,008	0	0	0	0.0000	0	0
A1 intron	465	0	0	0	0.0000		
intergenic A1-A2	4,822	0	0	0	0.0000		
A2 CDS	1,128	1	0	1	0.0009	1	0
A2 intron	640	0	0	0	0.0000		
intergenic A2-A3	5,393	0	1	1	0.0002		
A3 CDS	1,332	0	0	0	0.0000	0	0
A3 intron	1,396	2	0	2	0.0014		
intergenic A3-A4	18,521	4	5	9	0.0005		
A4 CDS	963	0	1	1	0.0010	0	1 (D/H)
A4 intron	546	1	0	1	0.0018		
intergenic A4-A5	11,081	8	5	13	0.0012		
A5 CDS	813	1	0	1	0.0012	0	1 (G/S)
A5 intron	960	1	1	2	0.0021		
intergenic A5-A6	2,030	1	1	2	0.0010		
A6 CDS	702	0	0	0	0.0000	0	0
A6 intron	1,383	1	0	1	0.0007		
intergenic A6-A7	7,201	3	2	5	0.0007		
A7 CDS	693	0	0	0	0.0000	0	0
A7 intron	917	1	0	1	0.0011		
intergenic A7-A9	4,879	5	1	6	0.0012		
sum	100,577	35	20	55	0.0005		
**Concatenated haploid sequence 2 (AB125655-AB125658) vs. NC_044609.1**							
intergenic A6-A7	3,076	0	0	0	0.0000		
A7 CDS	693	0	0	0	0.0000	0	0
A7 intron	932	0	0	0	0.0000		
intergenic A7-A9	7,049	2	4	6	0.0009		
A9 CDS	819	0	1	1	0.0012	0	1 (S/C)
A9 intron	1,036	0	0	0	0.0000		
intergenic A9-A10	6,456	3	4	7	0.0011		
A10 CDS	1,233	1	0	1	0.0008	0	0
A10 intron	1,175	1	1	2	0.0017		
intergenic A10-A11	8,483	6	4	10	0.0012		
A11 CDS	945	0	0	0	0.0000	0	0
A11 intron	1,399	1	0	1	0.0007		
intergenic A11-A13	13,035	7	4	11	0.0008		
A13 CDS	1,167	0	0	0	0.0000	0	0
A13 intron	713	0	0	0	0.0000		
intergenic A13-EVX1	42,931	37	14	51	0.0012		
EVX1 CDS	1,227	0	0	0	0.0000	0	0
EVX1 introns	2,177	0	0	0	0.0000		
3p non-genic	28,515	8	2	10	0.0004		
sum	123,061	66	34	100	0.0008		

ts: transition, tv: transversion, *s*: synonymous substitution, *n*: nonsynonymous substitution; *Amino acid differences are shown in parentheses.

## Abbreviations

NGS: next-generation sequencer
BAC: bacterial artificial chromosome
PCR: polymerase chain reaction
NCBI: National Center for Biotechnology Information
CDS: coding sequence
